# Yields and product comparison between *Escherichia coli* BL21 and W3110 in industrially relevant conditions: anti-c-Met scFv as a case study

**DOI:** 10.1186/s12934-023-02111-4

**Published:** 2023-05-19

**Authors:** Klaudia Arauzo-Aguilera, Luisa Buscajoni, Karin Koch, Gary Thompson, Colin Robinson, Matthias Berkemeyer

**Affiliations:** 1 School of Biosciences, University of Kent, Canterbury, CT2 7NJ UK; 2 Biopharma Austria, Process Science, Boehringer-Ingelheim RCV GmbH & Co KG, Dr. Boehringer-Gasse 5-11, 1121 Vienna, Austria; 3 Wellcome Trust Biological NMR Facility, School of Biosciences, University of Kent, Canterbury, CT2 7NJ UK

**Keywords:** Sec pathway, Fermentation, *Escherichia coli* BL21 and W3110, Disulphide bond, Product heterogeneity, Protein purification

## Abstract

**Introduction:**

In the biopharmaceutical industry, *Escherichia coli* is one of the preferred expression hosts for large-scale production of therapeutic proteins. Although increasing the product yield is important, product quality is a major factor in this industry because greatest productivity does not always correspond with the highest quality of the produced protein. While some post-translational modifications, such as disulphide bonds, are required to achieve the biologically active conformation, others may have a negative impact on the product’s activity, effectiveness, and/or safety. Therefore, they are classified as product associated impurities, and they represent a crucial quality parameter for regulatory authorities.

**Results:**

In this study, fermentation conditions of two widely employed industrial *E. coli* strains, BL21 and W3110 are compared for recombinant protein production of a single-chain variable fragment (scFv) in an industrial setting. We found that the BL21 strain produces more soluble scFv than the W3110 strain, even though W3110 produces more recombinant protein in total. A quality assessment on the scFv recovered from the supernatant was then performed. Unexpectedly, even when our scFv is correctly disulphide bonded and cleaved from its signal peptide in both strains, the protein shows charge heterogeneity with up to seven distinguishable variants on cation exchange chromatography. Biophysical characterization confirmed the presence of altered conformations of the two main charged variants.

**Conclusions:**

The findings indicated that BL21 is more productive for this specific scFv than W3110. When assessing product quality, a distinctive profile of the protein was found which was independent of the *E. coli* strain. This suggests that alterations are present in the recovered product although the exact nature of them could not be determined. This similarity between the two strains’ generated products also serves as a sign of their interchangeability. This study encourages the development of innovative, fast, and inexpensive techniques for the detection of heterogeneity while also provoking a debate about whether intact mass spectrometry-based analysis of the protein of interest is sufficient to detect heterogeneity in a product.

**Supplementary Information:**

The online version contains supplementary material available at 10.1186/s12934-023-02111-4.

## Introduction

*Escherichia coli* is one of the expression hosts of choice in the biopharmaceutical industry for large-scale production of therapeutic proteins because of its rapid growth, high product yield, cost effective production and easy scale-up processes [41]. If the protein of interest (POI) contains disulphide bonds (DSBs), as in the case of antibody fragments, periplasmic expression via the Sec pathway is often preferred [45]. Thereby the POI is transported in an unfolded state to the periplasm by fusing a signal peptide (SP) to the N-terminus of the POI. Once in the periplasm, correct DSB formation is achieved [23]. Furthermore, product translocation into the medium can be enforced which also simplifies downstream processing [58].

A good understanding of fermentation parameters and their impact on *E. coli* cell growth and final product yield is critical in defining biopharmaceutical production processes. Until today, many process adaptations to maximise product yield are based on optimizations of temperature, dissolved oxygen (DO) levels, pH, media composition, feeding strategies, etc. [26, 53]. However, maximum productivity does not always coincide with the highest quality of the recombinantly expressed protein [14]. Correct folding and in vivo stability of the recombinant protein are two crucial factors that must be controlled while optimising the cultivation conditions. Correct folding includes both the acquisition of the correct 3D structure as well as addition of post-translational modifications (PTMs), such as DSB formation. In *E. coli,* PTMs that occur during and after protein synthesis can represent a limitation when compared to other microorganisms. In the past decades several approaches have been explored in *E. coli* to overcome this drawback, reviewed by Rettenbacher et al. [41].

Some PTMs and physiochemical transformations of recombinant proteins can also originate from non-enzymatic reactions at all steps of the production process from cell culture to purification and storage [4]. In this case, PTMs are caused by chemical reactions occurring between the amino acid side chain and reagents present in either culture media or buffers in specific conditions of pH, temperature and oxygenation level. Some of these modifications can negatively affect the activity, efficacy and safety of the desired product by altering the product stability and its biological active conformation. Therefore, the percentage of product harbouring these modifications, within the heterogenous product pool generated, is identified as product related impurities and represents a crucial quality parameter for regulatory authorities [44]. Common PTMs are methionine oxidation, asparagine and glutamine deamidation, and aspartate isomerization. The importance of such unwanted modifications has been evaluated and ranked for recombinant monoclonal antibodies (mAbs) [29]. While N-terminal pyroglutamate, for example, is not considered as a critical quality attribute, modifications occurring in the complementary determining regions are of high importance as they could affect the antigen recognition capacity [29].

To investigate product heterogeneity, ion exchange chromatography [28, 34, 35] coupled with enzymatic digestion followed by mass spectrometry (MS) analysis (peptide mapping) [22, 49] are often the methods of choice. The former allows the separation of the protein heterogeneity based on the charge properties while the latter allows the identification of mass changes and the exact position of the modification within the protein expressed. However, since peptide mapping requires extensive work and can also generate artefactual modifications, the research for the improvement of this method is ongoing [10, 40].

In this study, fermentation conditions of two widely employed industrial *E. coli* strains, namely BL21 (B strain) and W3110 (K-12 strain) are compared for recombinant protein production of a single-chain variable fragment (scFv) in an industrial setting. Rather than analysing the well-known and studied performance and behavioural differences [31, 36, 47, 48], we focused on yields, and analysed the differences in product structure and heterogeneity between strains grown in 5 L fed-batch bioreactors using a number of different downstream and analytical techniques. The results reveal surprising difference in protein quantity and quality between the two strains, and equally surprising heterogeneity in the final preparations of this relatively simple biopharmaceutical.

## Results and discussion

### BL21 strain produces more soluble scFvM than W3110 strain

One of the major aims of this study was to directly compare the production of a biopharmaceutical product under industrial conditions in the two extensively used *E. coli* strains: BL21 and W3110. The viable and cost-effective production of a POI using *E. coli* varies enormously depending on many different factors. POI related factors and upstream process parameters such as pH, temperature, media composition, strain type and others influence the recombinant expression [25, 52].

Jones et al. [20] and Edwardraja et al. [8] have produced this scFv in *E. coli* in the periplasm after export by Tat pathway and expression in the cytoplasm, respectively, both at shake flask level. Traditional upstream bioprocess development involves the use of shaken bioreactor systems (usually shake flask). Cultivation in shake flasks is normally performed in a batch manner, provides very limited variable monitoring, and produces low cell densities and product yields. Furthermore, they rely on uncontrolled surface aeration leading to limited oxygen transfer rates and low batch-to-batch reproducibility [1]. Therefore, cultivation conditions that are used during shaken culture bioprocess development may be changed or completely discarded once they are optimized at pilot scale [39]. To overcome the limitations described above, there has been a concerted effort to develop fully automated high-throughput cultivation systems to significantly accelerate the identification of the optimal expression systems and process conditions [2, 16].

In our research, a screening of different conditions for the optimization of soluble yields of the scFvM was carried out for both strains listed above combining different temperatures, pH and inducer concentrations (described in “Materials and Methods”). This screening showed only minimal changes in OD_550_ and titer. However, a further optimization of media composition and induction time could not be carried out since the implementation of these changes would require a complete revaluation of this automated protocol for 10 mL fermentation [16].

**Fig. 1:**
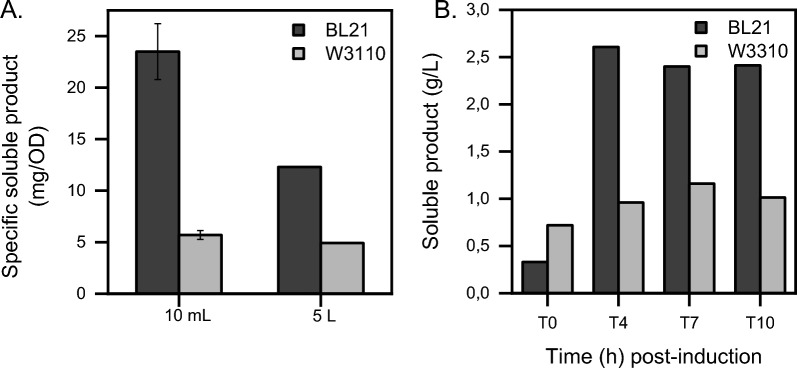
Soluble scFvM production in BL21 and W3110 in 10 mL and 5 L scale fermentations. **A** Specific soluble product titer in BL21 and W3110 in 10 mL and 5 L bioreactors expressed in mg/OD. To calculate these values, the 7 h post-induction soluble scFvM titer is divided by the OD_550_ of the culture at that time in both scale bioreactors. 10 mL fermentations specific soluble product formation values are the average of four replicates, error bars have been added to the figure. **B** Soluble titer variation of the scFvM in BL21 and W3110 strains at different time points [0 h (T0), 4 h (T4), 7 h (T7) and 10 h (T10) post-induction] in 5 L fermentations expressed in g/L. In both graphs, BL21 strain is represented in black and W3110 in grey. Soluble scFvM product was determined by immunoassay from suspension samples. 5 L bioreactors were run two times for each strain with slight variations in temperature, inductor concentration and pH resulting in comparable profiles for OD_550_ and titer

In Fig. 1A, the specific soluble product formation was calculated for BL21 and W3110 in 10 mL and 5 L bioreactors following the standard protocol at 7 h post-induction (T7) (refer to “Materials and Methods”). To eliminate a possible impact of the different optical density (OD) levels at both scales, the specific soluble product titer was determined by dividing soluble product titer by OD_550_ for both strains and scales at time point T7 (end of fermentation in 10 mL scale) (Fig. 1A). The BL21 strain shows a specific soluble product titer of 23.5 mg/OD in 10 mL fermenters and 12.3 mg/OD in benchmark 5 L fermenters. Janzen et al. [16] also described a higher specific soluble product titer formation in small‐scale cultivations than in 5 L fermenters when employing a B strain. It has been suggested by Kang et al. [21] that BL21 may suffer from DO limitations in large-scale cultures, however this hypothesis could not be verified by our research due to the absence of comparison data between the two fermentation scales. The W3110 strain, on the other hand, shows very similar specific soluble product titers in both scales: 5.7 mg/OD in 10 mL fermenters and 4.9 mg/OD in 5 L fermenters. These results validate the robustness and reproducibility that this strain provides in industry [21, 56]. However, with respect to the expression strains used, BL21 showed significantly higher titers in all direct comparisons (Fig. 1A). Specific soluble product titer comparison between scales was also carried out with the optimized conditions after the screening experiments in 10 mL fermenters (refer to “Materials and Methods”). Since a very similar pattern of soluble protein was obtained when comparing scales and strains, the data set is not shown because it had a comparable trend.

Looking more closely at the benchmark process, Fig. 1B shows the soluble production of scFvM in BL21 and W3110 in 5 L fermenters and standard conditions at different time points: 0 h (T0), 4 h (T4), 7 h (T7) and 10 h (T10) post-induction. Soluble scFvM was quantified by an immunoassay from suspension samples. Overall, BL21 shows a higher soluble protein content (two-fold) during the entire induction period in the 5 L fermentation system compared to W3110 (Fig. 1B). In BL21, the peak production of soluble protein is achieved 4 h after induction (2.61 g/L) and it remains stable until T10 (end of fermentation, 2.41 g/L). BL21 shows a tight regulation of the tac promoter under non-induced conditions (0 h post-induction): it leaks 0.33 g/L. On the other hand, W3110’s peak production of soluble protein is achieved 7 h after induction (1.16 g/L) and it also remains stable until T10 (end of fermentation, 1.01 g/L). Unlike BL21, W3110’s tac promoter is leakier and produces more than half of the total soluble scFvM before induction (0.72 g/L). This leaky expression in W3110 could be linked to plasmid instability, which many times explains a poor yield of target protein [43]. However, in this case, differences in yield between the chosen strains are not connected to plasmid loss or instability, as plasmid copy number (PCN) remains stable and comparable between them throughout the whole fermentation process (observed: ≈ 12–18 copies/cell,expected: 15–20 copies/cell). When plasmid instability is discarded, BL21 and W3110 critical genome differences for recombinant protein production should be considered to understand these yield differences. Even though BL21 and W3110 are both widely used in recombinant protein production, B strains are deficient in the Lon protease, which degrades many recombinant proteins. The B strain also lacks the outer membrane protease OmpT, whose function is to degrade extracellular proteins [43]. These genetic differences between strains may explain the higher yields obtained with BL21. In addition, it should be noted that BL21 reaches a lower OD_550_ value than the W3110 strain (BL21: 212 and W3110: 272) at the end of fermentation. These OD differences between the compared strains might correlate with the metabolic burden caused by the continuous export to the periplasm by Sec pathway [13] and/or the lethal outer membrane punctures occurred as a result of limited periplasmic capacity [46, 51] in BL21. A second run of bioreactors with parameters optimised for BL21 (refer to “Materials and Methods”) was carried out and similar patterns for yields and OD_550_ values were obtained compared to standard conditions (data set is not shown because it had a comparable trend).

**Fig. 2:**
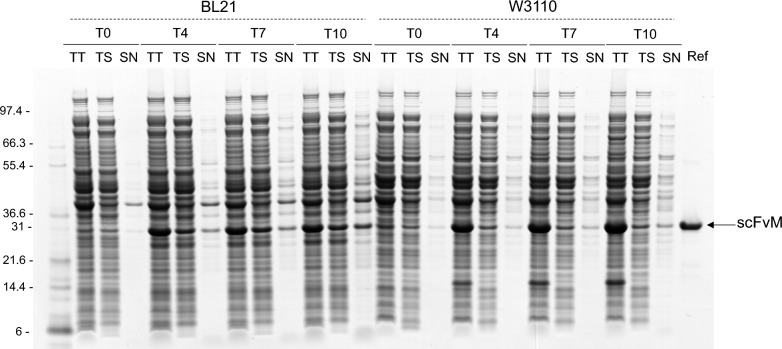
Lysates of BL21 and W3110 from 5 L fermenters when expressing OmpA-scFvM. Suspension and supernatant samples of BL21 and W3110 expressing OmpA-scFvM were recovered at different time points: T0, T4, T7 and T10 for SDS-PAGE analysis in reducing condition. Representative Coomassie blue-stained gel of the total titer (TT, comprising soluble and insoluble proteins), total soluble (TS, comprising intracellular and extracellular soluble proteins) and supernatant samples (SN, only proteins located in the supernatant) and scFvM reference protein.Ladder (Mark12^™^ Unstained Standard) on the left in kDa. The same volume of sample was treated and loaded for comparison. OD of the samples: BL21: T0 (167); T4 (229); T7 (231); T10 (212) and W3110: T0 (165); T4 (217); T7 (236); T10(272)

Even when the focus is on soluble production, an additional inherent part of disulphide bonded protein production in *E. coli* cannot be dismissed: inclusion body (IB) formation. The Coomassie blue-stained gel in Fig. 2 shows the lysates of BL21 and W3110 from 5 L fermenters when expressing OmpA-scFvM at T0, T4, T7 and T10 time-points in standard conditions. Cell suspension was analysed and fractionated in total titer (TT, comprising soluble and insoluble proteins), total soluble (TS, comprising intracellular and extracellular soluble proteins) and supernatant samples (SN, only proteins located in the extracellular medium). The insoluble POI production is remarkably different between the compared strains (Fig. 2). When looking at the Coomassie blue-stained gel, it is important to notice that total production of the POI (TT) is visually higher in W3110 than in BL21 at all time points. This result suggests that the majority of the protein is produced as IBs and only a small part is translocated to the periplasm and extracellular medium and is therefore soluble (TS). As explained before in Fig. 1B, and as it can be noticed in the Coomassie blue-stained gel in Fig. 2 (see and visually compare T0 scFvM production in both strains), W3110 pre-induction leakiness is higher than BL21’s. We hypothesise that due to this early high-level expression in W3110, hydrophobic stretches in the polypeptide are present at high concentrations very early in the cell and are available for interaction with similar regions. This may lead to protein instability and aggregation (IB formation) [6, 43]. Over time, and in both strains, but more remarkably in BL21, the POI starts to be detectable in the supernatant due to the leakiness of the outer membrane [46, 51], active export [55] and/or lysis of the cells [24].

### The purified scFvM shows multiple charged variants on a CEX

**Fig. 3:**
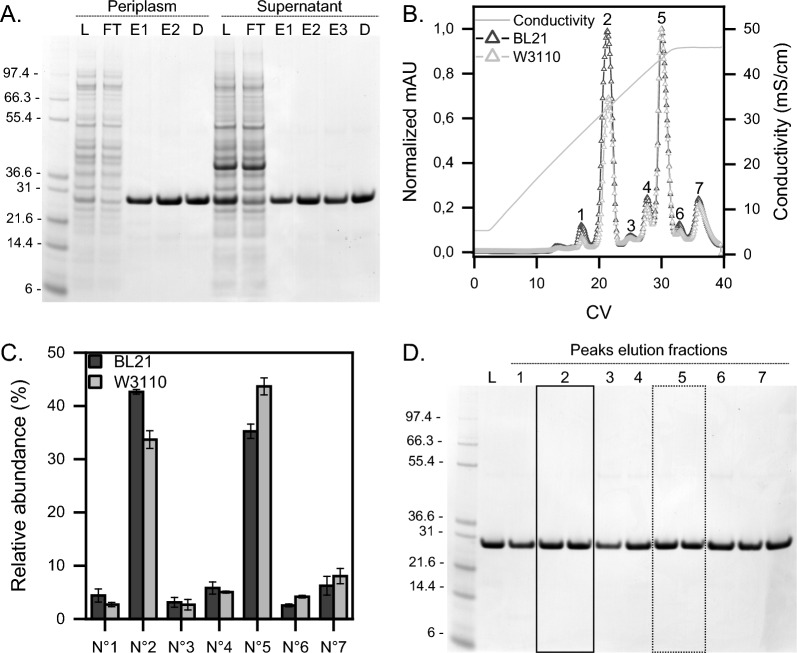
scFvM purification via two steps chromatography. **A** Representative Coomassie blue-stained gel of the scFvM produced with BL21 and purified via IMAC. The samples analysed are the total protein sample loaded (L), column flow-though (FT), eluates (E) from either the periplasm or the culture supernatant and the purified protein after dialysis (D) in non-reducing condition. Ladder (Mark12^™^ Unstained Standard) on the left in kDa. **B** Representative MonoS normalised chromatograms of scFvM purified from the culture supernatant. Peaks are labelled with numbers ranging from 1 to 7. **C** Relative abundance of the CEX peaks in BL21 and W3110. BL21 strain is represented in black and W3110 in grey. **D** Representative Coomassie blue-stained gel of a CEX run in non-reducing condition on the scFvM produced with the BL21 strain. peak N°2 elution fractions are in the continued box while peak N°5 ones are in the dotted box

The second objective of this research was to determine whether the protein expressed by the two strains, after translocation in the periplasm, was similarly folded and contained the same charge heterogeneity. The produced scFvM from the T10 (end of fermentation) was purified from periplasmic extract and from culture supernatant by nickel immobilised metal affinity chromatography (IMAC). Table 1 shows the amount of soluble protein obtained after purification of the same volume of either supernatant or extracted periplasm. It should be noted that the intracellular soluble titer (periplasmic fraction) was higher than the extracellular one in both strains. However, this was not reflected in the product titer after purification. This difference depends on the low amount of periplasm that could be extracted due to setting constraints in the maximal cell pellet that can be processed (“see Materials and Methods”). Analysis via Coomassie blue-stained gel (BL21 Fig. 3A, W3110 SI Fig. 1A) shows that the scFvM was obtained with high purity, independently of the expression host and purified compartment.

**Table 1:** Comparative analysis of the scFvM produced by the two strains in the different compartments

Strain	Location	Protein concentration (g/L)	Protein purified (mg)	Purification yield (%)	Purification factor^a^	Purity (%)
BL21	Periplasm	2.1	0.5	51.8	7.1	92.2
	Supernatant	0.5	45.9	81.4	3.7	91.5
W3110	Periplasm	0.9	0.3	77.5	9.4	91.3
	Supernatant	0.2	26.3	99.6	7.2	92.3

^**a**^The purification factor was calculated dividing the purification yield (%) over the relative abundance (%) of the target protein in the examined compartment

To further investigate the presence of possible heterogeneity of the expressed scFvM, the IMAC purified material from culture supernatants was separated by cation exchange chromatography (CEX) via gradient elution, since the scFvM has a basic isoelectric point of 7.8 (Expasy Protparam). CEX is typically considered a gold standard technique to separate and purify charge variants [54, 57]. However, the results from this technique can be strongly influenced by differences in operational parameters such as column type, particle size and flow rate [9, 18]. Since previous studies demonstrated the importance of the diameter resin particles and flow rate on the separation performance [18, 57], a small resin particle (10 µm diameter resin) coupled with a slow flow rate (0.5 mL/min) was selected in our case. The results indicated a high separation performance. The chromatograms show the presence of two main peaks: one more acidic (N°2) and another one more basic (N°5), both coupled with some minor subforms (Fig. 3B). Between the two strains, the elution pattern is maintained, however, the relativity of the peaks slightly changes BL21 being richer in acidic variants while W3110 in basic ones (Fig. 3C). To verify, firstly, if the multiple peaks showed different masses and impurities, a non-reducing gel was assessed. However, no differences could be detected (BL21 Fig. 3D, W3110 SI Fig. 1B).

### scFvM is correctly disulphide bonded and cleaved from its SP in BL21 and W3110

In this study an offline approach was applied, consisting in the isolation of the separated forms from CEX followed by individual analysis for better understanding of possible modifications. The workflow involved coupling size-exclusion chromatography (SEC) directly to mass spectrometry (MS). This was done to verify that the scFvM was correctly folded and that different peaks on CEX were not caused by a pool of species with free thiols or uncleaved SP. IMAC and CEX purified samples (peaks N°2 and N°5) from both periplasm and supernatant samples from BL21 and W3110 were analysed by LC–MS. This analysis confirmed that all scFvM samples had the expected molecular weight, consistent with the cleavage of the SP when the POI is exported from the cytoplasm to the periplasm and its four cysteines in two DSBs (Table 2). The main component in all these samples is the unmodified scFvM molecule. In addition to it and as second species, both BL21 and W3110 samples show comparable reduction of −17 Da, probably due to N-terminal pyroglutamate modification (pyroQ), while gluconoylation is only seen in BL21 samples (+ 178 Da). PyroQ modification is generated after a non-enzymatic cyclization of N-terminal glutamine whose rate of formation can be affected by various environmental factors during purification and storage [3, 4]. In previous studies, CEX has been reported as a method of choice for the separation of pyroQ modifications since the loss of a primary amine causes an acidity shift of the antibody [4, 5]. However, in this study the use of a strong cation exchange did not show the same results. In fact, LC–MS analysis run on each of the CEX peaks showed the presence of a −17 Da modifications, ranging from 12–25%, in each sample (Table 2). The strain selectivity of the non-enzymatic gluconoylation modification agrees with previous literature. B strains accumulate 6-phosphogluconolactone due to the lack of 6-phosphogluconolactonase [33], which favours gluconoylation, so it is not unexpected that this strain produces some gluconoylated proteins. Although this modification can adversely affect protein quality, the gluconoylation is not very stable and can transform back into unmodified protein and gluconate via a hydrolysis reaction [32].

**Table 2:** Analysis of free thiol content, SP cleavage and secondary modifications based on MS

Strain	Technique	Location	N° of Cysteine	M_ox Theor_^a^	M_Exp_^b^	Δ mass	+ 178 Da (5–11%)	−17 Da (12–25%)
BL21	IMAC	Periplasm	4	27478.27	27478.27	0	+ ^c^	+
		Supernatant		27478.27	27478.27	0	+	+
	CEX	Peak N°2		27478.27	27478.27	0	+	+
		Peak N°5		27478.27	27478.27	0	+	+
W3110	IMAC	Periplasm		27478.27	27478.27	0	−	+
		Supernatant		27478.27	27478.27	0	−	+
	CEX	Peak N°2		27478.27	27478.27	0	−	+
		Peak N°5		27478.27	27478.27	0	−	+

^**a**^Calculated theoretical oxidised molecular weight (M_OxTheor_)

^**b**^Experimental molecular weight (M_Exp_)

^**c**^The sign “ + ” indicates the presence of the modification (+ 178 Da or −17 Da) while “−” indicates the absence of it

In addition, possible mismatches of the DSBs were also analysed among the multiple peaks in CEX (purified from BL21) by MS. In this case the purified protein from each CEX peak was digested. However, no differences in the size of the peptides generated were identified both in native conditions and after reduction, confirming that DSB shuffling is not essentially the reason for the heterogeneous pattern in CEX (Table 2).

### The stability of the two main peaks excludes a handling artefact

**Fig. 4:**
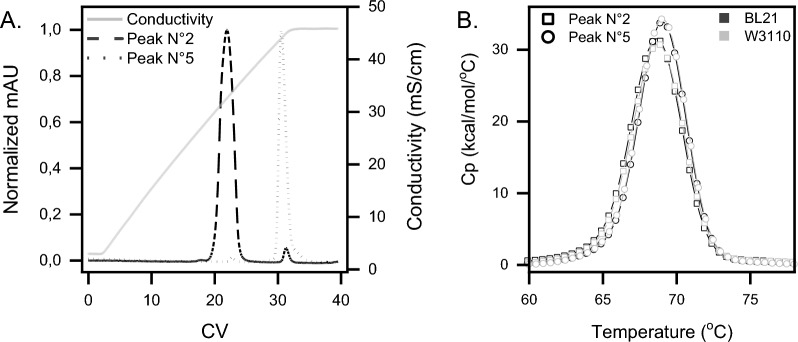
Stability analysis of the two main peaks from the CEX. **A** Overlaid MonoS chromatograms of the two main peaks that were collected, dialyzed, and reloaded separately to demonstrate the stability and purity of the two forms. **B** DSC profiles of the peaks N°2 and N°5 obtained from CEX purification of scFvM expressed by BL21 and W3110. The obtained Tms are peak N°2 68.7 ℃ and peak N°5 69.1 ℃. Squares represent peak N°2 and circles peak N°5. BL21 strain is represented in black and W3110 in grey

A reversibility analysis on the two main peaks was then performed to verify if these two main CEX forms were not an increasing modification caused by downstream operation. The eluted single peaks were therefore pooled from different purifications in N°2 and N°5 respectively, dialyzed against the equilibration buffer and each pool was loaded again on the CEX. Figure 4A shows the comparison of the elution profiles from each pool. In both cases the purification revealed a perfect reproducibility of the peaks that seem to be only in a very slow reversible equilibrium with the counterpart. Moreover, since with this experiment the heterogeneous pattern observed during the CEX purification of the purified material (Fig. 3B) was not visible, an artefact of the column caused by overloading of the samples could be excluded. The two peaks therefore represent scFvM isoforms that are stable under these conditions.

### Biophysical characterization confirmed altered conformations of the two main charged variants

To further investigate potential conformational differences between the two main CEX forms, and the exact comparability among the two strains, a biophysical assessment was established via differential scanning calorimetry (DSC) and one-dimensional proton nuclear magnetic resonance (1D-^1^H-NMR) spectroscopy. DSC is commonly employed to assess the thermal and conformational stability of a protein under specific buffer conditions [19]. The two separated main peaks (N°2 and N°5) were evaluated by DSC (Fig. 4B). One single transition, corresponding to the unfolding of the scFvM, was observed in all samples. The melting temperatures of the single peaks N°2 and N°5 (from both BL21 and W3110) were 68.7 ℃ and 69.1 ℃, respectively and corresponds to a temperature difference of 0.4 ℃. These results proved a high comparability, in terms of thermal stability, of the POI produced by the two *E. coli* strains.

**Fig. 5.**
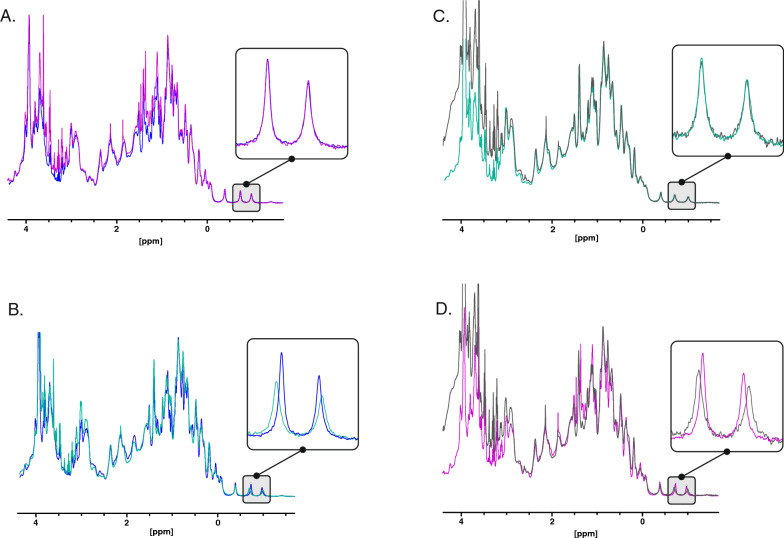
^1^H-NMR spectra of peaks N°2 and N°5 conformers measured at 600 MHz. Spectra from basic, acidic species and control samples show the conformational and temporal stability of the species. The samples were exhaustively co-dialysed before analysis. Samples conditions: pH 5.5 and 25 ℃, sample concentration ~ 50 µM. **A** Spectra of peak N°2 from BL21 (purple) and W3110 (blue). **C** Peak N°5 from BL21 (grey) and W3110 (green) representing analysis of the same species showing reproducible conformational state as a control and spectra showing the clear difference in the fingerprint of peak N°2 and N°5 conformers from W3110 (**B)** and BL21 (**D**). The box represents the expansion of the high field methyl aromatic fingerprint region

In addition to this thermal stability difference, a modification in the state of the protein from the two main peaks from the CEX was detected via one-dimensional proton ^1^H-NMR spectroscopy (1D-^1^H-NMR). This technique shows signals for each hydrogen atom in the protein that is covalently bound or exchanging slowly with water (for example amide signals will be present but those from –OH and NH_3_ groups will be missing). These signals resonate at different frequencies (chemical shifts in ppm; parts per million of the main field) and with different intensities based on the 3D structure, ligand binding state and dynamics of the protein all of which affect local magnetic fields in the protein. The position of peaks in the 1D-^1^H-NMR depend at first order on the chemistry of the atoms [27], so for example CH_2_ and CH_3_ groups from different amino acid types (e.g., Val vs Leu) appear at different positions and also have small differences due to the primary sequence. On top of these chemical effects from residue types and the primary sequence the spectrum is also extremely sensitive to the 3D structure of the protein and very small changes in local environment and dynamics (see for example [7] can be detected, so 1D-^1^H-NMR can be used as a fingerprint of the proteins 3D structure and to monitor small changes in the state of the protein. In this case the spectra suggest both samples contain proteins that are well-folded as indicated by the well resolved and dispersed signals in the amide region (not shown) and a series of well-resolved methyl peaks at < 0 ppm which are indicative of stable methyl aromatic packing in the protein's core (boxes in Fig. 5). On top of this, the spectra are similar enough to conclude that there have been no major changes in 3D structure and that the overall 3D fold is the same. However, while the methyl peaks at < 0 ppm are well dispersed, they also show variations between the two CEX peak samples (N°2 and N°5). For CEX peak N°5 the methyl signals that resonate at −0.736 ppm and −0.981 ppm show shifts of + 0.04 ppm and ~ −0.02 ppm and ~ 10–20% weaker peak height than CEX peak N°2 for both strains (Fig. 5B and D). As the samples were very thoroughly dialysed these differences would indicate a change in conformation within the core of the protein or the presence of a yet unidentified strongly bound ligand.

All these results combined suggested that the two strains produced a target protein with the same overall 3D fold and thermal stability. However, the difference between the two main peaks of the CEX seems to be generated by a conformational change of the protein that leaves the mass unchanged as observed by MS analysis. Two main reasons could be behind this result: the presence of a ligand tightly bound to the protein or a modification that leaves the mass unchanged. With regards to the ligand, the reversibility analysis on the two main peaks suggests that, if present, the ligand binds with high affinity to one of the two forms since it could not be removed during the dialysis process. Moreover, at least, the presence of a high molecular mass molecule as ligand could be excluded since, prior LC–MS analysis, the samples were desalted by size exclusion chromatography (SEC) and no additional peaks were observed. Concerning the second hypothesis, various types of modification can occur during protein expression, manufacturing, and storage [3, 4]. Some of these modifications can lead to mass shifts while some others can result in protein modifications leaving the mass unchanged. Examples of the latter are DSB mismatch and aspartate isomerization which can further generate aspartic acid racemization [54]. The presence of mismatched DSBs was excluded as a possible reason for multiple peaks in CEX, as described above. Aspartate (Asp) isomerization is a non-enzymatic modification that can cause conformational changes of the protein since it introduces an additional methyl group in the protein backbone [3, 4]. Furthermore, the specific structural outcome can lead to two isomeric products (l-isoAsp and d-isoAsp) where the D-amino acid can affect the peptide function [42]. This reaction occurs at an optimal pH of 5, produces succinimide (−18 Da specie) as a reaction intermediate and it is favoured on aspartate residues that are followed by a glycine [11, 37, 38]. Moreover, it was shown in a previous study that antibody variants containing isoaspartate elute later in CEX [12]. The current analysis was carried out at pH 5.5, a second and more basic peak was present, in the protein sequence an aspartate close to a glycine is present and a species with −17/18 Da was detected. Therefore, the presence of aspartate isomerization as a possible modification cannot be excluded completely. There are some other methods to identify this modification such as LC–MS peptide mapping and 2D NMR analysis. LC–MS peptide mapping is becoming an important method for the characterization of primary sequences and PTMs in antibody products. On the other hand, this technique is labour-intensive, time-consuming and can introduce artificial PTMs, resulting in an overestimation of the target protein modifications. During sample preparation, long digestions can generate unnecessary reactions interfering with the quantitation of the peaks of interest, causing low reproducibility of the results. Shortening digestion-time can cause incomplete peptide cleavages, thus low sequence coverage and poor repeatability [17]. In this case study, where BL21 and W3110 protein product comparison was the aim, this technique had to be discarded due to reproducibility issues. On the other hand, 2D NMR analysis typically utilises labelled isotopes during the fermentation process for proteins of this mass, and therefore, this approach was not available due to experimental constraints.

## Conclusion

In this case-study, we report that *E. coli* strain differences may have an influence on the final product yield but not necessarily on its heterogeneity pattern. The results showed that BL21 and W3110 have a very different productivity profile in the conditions employed, with BL21 being more industrially relevant to produce this specific scFv in terms of yield. In terms of quality, except for the B strain characteristic gluconoylation, the expressed scFvM displays a similar heterogeneity profile. This resemblance of the produced product represents an indication of the interchangeability between the two strains, a characteristic that presents an important perspective for biosimilar and biobetter production. In our paper it is further shown that when scFvM product quality was assessed by different analytical methods, a distinctive profile of the product was obtained, suggesting that alterations in the recovered product are present independently of the host strain. These alterations appear to be stable and significant, since the two forms elute at very different salt concentrations during CEX. However, the exact identity of the cause of product heterogeneity could not be appropriately confirmed. This opens a discussion on whether MS based intact mass analysis of the POI is enough to spot the heterogeneity in a product. At the same time, we want to encourage the discoveries of new, fast and affordable methods for analysis of heterogeneity other than 2D NMR and LC–MS based peptide mapping for the identification of protein heterogeneity in biopharma, which are time-consuming.

## Materials and methods

All chemicals, reagents and enzymes were of highest quality and were obtained from Sigma-Aldrich, Roth or Thermo Fisher Scientific, unless otherwise noted.

### anti-c-Met scFv (scFvM) expression strain generation

*Escherichia coli* DH5α (Invitrogen) was used for genetic manipulations. The anti-c-Met scFv (sequence taken from Edwardraja et al. [8]), with an N-terminal wild-type OmpA SP and a C-terminal 6 × His-tag was commercially synthesised (GeneArt). The synthesis construct was sub-cloned into the pFLAG-CTC vector (Sigma Aldrich) under the control of a tac promoter using NdeI and EcoRI restriction sites. This construct will be termed as OmpA-scFvM in this work. Individual clones were sequenced before transforming the expression plasmid into the expression strains *E. coli* BL21 (Novagen) and W3110 (DSMZ). In this work, the protein anti-c-Met scFv is referred to as scFvM to further correlate with Edwardraja et al. and Jones et al. [8, 20] which investigated the same protein in a different setup.

### Expression in a miniaturised fermentation platform (Multifermenter, MF) and a 5 L fermentation system

The fully automated cultivation at 10 mL scale in the MF was performed as described in Janzen et al. [16] with the exception that the temperature in all reactors was set to 37 ℃ and lowered to the corresponding experiment temperature prior to induction. For the screening of conditions, a range of different temperatures (25–33.5 ℃), pH values (6.3–7.3) and isopropyl β-d-1-thioglactopyranoside (IPTG) inducer concentrations (0.5–1 mM) were tested with a design of experiment (DoE) setting in 32 MF vessels. While for BL21 a slight increase in yields and OD was observed in a screened condition set-up (refer as optimised conditions), with W3110 no optimisation could be achieved. Therefore, solely the standard conditions developed by Janzen et al. [16] and optimised conditions developed for BL21 after MFs run were tested in quadruplicates in 10 mL bioreactors for BL21 and W3110 to maintain a better comparability within the study. In the standard set-up, temperature was set to 30 ℃ prior to induction and the pH was constantly maintained at 6.8. Cultures in this case were induced with 1 mM IPTG (0.024 mL from 75 mM stock). In the optimised BL21 set-up, temperature was set to 32 ℃ prior to induction and the pH was constantly maintained at 7.3. Cultures in this set-up were induced with 0.5 mM IPTG (0.012 mL from 75 mM stock). In all cases, the DO level was maintained at ≥ 35%. In the case of the W3110 strain, a pre-culture in shake-flask was performed, since a significantly prolonged batch phase (e.g. lag phase of the cells) interfered with the fermentation protocol in the MF. The pre-culture was performed at 37 ℃ and 250 rpm until the culture reached an OD_550_ value of 2. The OD_550_ measurement of the pre-culture was manually performed (Genesys 10S UV‐Vis; Thermo Fisher Scientific). In the case of BL21, bioreactors were directly inoculated from the cell bank.

Benchmark fed‐batch cultivations were performed in fully controlled 5 L standard stirred‐tank bioreactor systems (BIOSTAT Cplus; Sartorius Stedim) and the manufacturer provided PCS (MFCS‐Win; Sartorius Stedim). Calibration and cultivation conditions and the used material and equipment are described in Janzen et al. [16]. As in MF experiments, the two set-ups (standard conditions, data shown in Results and Discussion section and optimised for BL21, data set not shown as it had a comparable trend as standard conditions) of the experiments were carried out with slight differences in the temperature, pH and induction concentration parameters (more details above). In all cases, the DO level was maintained at ≥ 35% and the pH was kept constant at the set pH ± 0.2 using 25% ammonia and 3 M phosphoric acid. Cultures were induced either with 0.5 mM (optimised conditions for BL21) or 1 mM IPTG (standard conditions) (11.8 mL or 23.6 mL from 211.9 mM stock, respectively). Samples for product quantification, PCN estimation and OD_550_ determination were manually withdrawn before induction (T0) and 4 h (T4), 7 h (T7) and 10 h (T10, end of fermentation) after the IPTG pulse. OD_550_ measurements were directly performed (Genesys 10S UV‐Vis; Thermo Fisher Scientific). Samples for product quantification and PCN estimation were stored in reaction tubes at—20 ℃ until further use.

The minimal media used for all cultivations (both 10 mL and 5 L systems) were prepared with potassium phosphate as buffering agent and source of P and K. Moreover, it contained trisodium citrate, MgSO_4_, CaCl_2_, glucose and the trace element solution. The trace element solution and the concentrations of additives used were the same as the ones described in Striedner et al. [50]. The medium was further supplemented with 1 mL/L antifoam agent (PPG 2000,Dow Chemical Co.) and was autoclaved for sterilization in place (SIP) prior cultivation in case of the 5 L system. In the MFs, each block was equipped with bioreactors which were aseptically filled with sterile medium supplemented with 1 mL/L antifoam agent (PPG 2000; Dow Chemical Co.) in a laminar flow hood.

### Fermentation sample preparation and quantification

Quantitative analysis was performed by automated immunoassay (Gyros). The sample preparation was conducted with a liquid handling system (Tecan) in 96-well format. For fermentation suspension samples, high viscosity due to leaked nucleic acids caused by fermentation condition and sample freeze and thawing was encountered. High viscosity causes imprecise pipetting by the liquid handling robot. Therefore, as an initial step a nucleic acid hydrolysis with Benzonase^®^ (Merck) (0.5 U/μL for ≥ 10 min, 450 rpm at room temperature (RT)) was performed. Cell lysis was performed by incubation with 1/10 v/v Lysonase (Merck) in FastBreak cell lysis reagent (Promega) for 30 min at RT with shaking at 450 rpm. The soluble fraction was analysed by the immunoassay. For this purpose, the digested cells were centrifuged at 2900 g for 10 min (Tecan centrifuge Hettich Rotanta) and the supernatant was further used. Samples were diluted in the analysis buffer (RexxipA (Gyros)) for quantification.

In the case of supernatant samples from fermentation, straight dilution in the analysis buffer (RexxipA (Gyros)) was performed.

Content quantification was performed using a Gyrolab xPlore by an automated immunoassay with an scFv-specific antibody (109-066-097 (Jackson ImmunoResearch), biotinylated for immobilization within the Gyros CD‐microstructure) and an his-tag specific antibody (34670 (Qiagen), Alexa647 fluorescence labelled for detection). The Gyros protocol (200‐3W‐002‐A) was performed according to the manufacturer’s instructions. The standard curve was analysed with the Gyros Evaluator SW using a five‐parameter fit. Quantification was performed in the linear range of the standard curve (15 ng/mL to 1000 ng/mL).

### Plasmid copy number (PCN) estimation

For PCN estimation, 5/OD_550_ fermentation pellet samples were used. Fully automated plasmid extraction was performed using the QIAprep Spin Miniprep Kit (Qiagen) and the QIAcube Connect (Qiagen). DNA concentrations were measured using a NanoDropTM (Thermo Fisher Scientific). The following equation was used to estimate the PCN:

$$PCN=\frac{Plasmid \,concentration \left[\frac{ng}{\mu L}\right] * 30 \,\mu L * 1.32 (correction \,factor\, for \,plasmid \,loss)}{nucleotides \,of \,plasmid * 308.95 \frac{Da}{nucleotide} * \left(1.66* {10}^{-15}\frac{ng}{Da}\right) * (7.4* {10}^{10}\frac{cells}{pellet})}$$

The weight per single plasmid in ng was calculated from the number of nucleotides and the conversion factors 308.95 (mean weight per nucleotide in Da) and 1.66∙10^–15^ (conversion from Da to ng). DNA loss during plasmid preparation (determined by spiking experiments) and the average number of cells in 5/OD_550_ pellet samples (7.4∙10^10^, determined in a cell counting chamber (Marienfeld Superior)) were considered to calculate the number of plasmids per single cell.

### Isolation of scFvM from periplasm and supernatant and content quantification

The expressed scFvM was purified from supernatant and periplasm. The periplasmic extraction was achieved following the pureFrac protocol as described elsewhere [30]. Since a larger volume was necessary an upscaling factor of 130 was applied during all steps. The periplasmic fractions and the supernatant from BL21 and W3110 were then applied on a Ni SepharoseTM 6 Fast Flow (Cytiva) column on the ÄKTA Avant chromatography system (GE Healthcare) at RT and purified via IMAC. At a flow rate of 1 mL/min low binding impurities were washed from the column with 10 column volumes of equilibration buffer (20 mM Phosphate, 500 mM NaCl, 20 M Imidazole, pH 7.4). The protein was then eluted with 5 column volumes of 100% elution buffer (20 mM Phosphate, 500 mM NaCl, 500 mM Imidazole, pH 7.4). The protein content was monitored online by absorbance at 280 nm. After each purification the column was stripped, washed, and recharged to avoid contaminations between the different strains and purified compartments. The eluted protein was dialyzed against 20 mM Phosphate, 20 mM NaCl, at pH 5.5 using 3.5 molecular weight cut-off (MWCO) dialysis cassettes (Thermo Scientific) and stored at −20 ℃ between purification steps.

With the eluted and rebuffered fractions (20 mM Phosphate, 20 mM NaCl, pH 5.5) from the supernatant a CEX with a MonoSTM 5/50 GL (Cytiva) column was performed on an ÄKTA Purifier chromatography system (GE Healthcare) at RT. The used buffers were 20 mM Phosphate, 20 mM NaCl, at pH 5.5 (equilibration buffer) and 20 mM Phosphate, 500 mM NaCl, at pH 5.5 (elution buffer). At a flow rate of 0.5 mL/min, a gradient from 0–100% elution was applied for 30 column volumes followed by 10 column volumes at 100% elution buffer. The protein content was monitored online by absorbance at 280 nm. The eluted protein was dialyzed against 20 mM Phosphate, 20 mM NaCl, at pH 5.5 using 3.5 MWCO dialysis cassettes (Thermo Scientific) and concentrated with 3 kDa MWCO Pierce™ Protein Concentrators PES (ThermoFisher). The fractions were stored at −20 ℃ until analysed.

Purified protein concentration was determined by measuring the absorbance at 280 nm with the Nanodrop 2000 (ThermoFisher) and a calculated molar extinction coefficient of 58′580 M^−1^ (Expasy Protparam). Affinity purified scFvM and its charged variants (CEX samples) were denatured for 5 min at 80 ℃ and run on an SDS-PAGE gel under reducing and non-reducing conditions.

### DSC analysis

The DSC experiments were performed using a MicroCal VP-DSC system (Malvern). All samples were dialyzed against the same buffer (20 mM Phosphate, 20 mM NaCl, pH 5.5) prior analysis using 3.5 MWCO dialysis cassettes (Thermo Scientific). The reference cell was filled with a buffer corresponding to the sample buffer. The samples were heated from 10 ℃ to 95 ℃ at a heating rate of 60 ℃/h. The pre-scan was 3 min, the filtering period was 10 s, and the feedback mode/gain was set to passive. The midpoint of thermal transition temperature (Tm) was obtained by analysing the data using OriginTM 7 software. All experiments were performed at a protein concentration of 1 mg/mL.

### Mass spectrometry analysis

For intact mass analysis, samples were injected without prior sample preparation into the UltiMate^™^ 3000 UHPLC system coupled to the Orbitrap Eclipse^™^ Tribrid^™^ mass spectrometer (all Thermo Fisher Scientific). Proteins were loaded on an ACQUITY UPLC BEH200 SEC, 1.7 µm, 4.6 × 150 mm, applying a 10 min isocratic method (20% B), with a flow rate of 0.2 mL/min and 0.1% Formic acid (FA) (Fisher Chemical, LC–MS grade) in water as mobile phase A and 0.1% FA in Acetonitrile (ACN) (Merck, Hypergrade for LC–MS) as mobile phase B. Electrospray ionisation was performed in positive ionisation mode and molecules analysed in the Orbitrap with a scan range of 500–2000 m/z and a resolution set to 240 000 (at 200 m/z) for full scan.

For DSB analysis, proteins were precipitated with CHCl_3_/Methanol, dried at RT and subsequently dissolved in lysis buffer (7.6 M urea/50 mM Tris–HCl, pH 8), diluted with 50 mM Tris–HCl, at pH 8 and digested with Trypsin/Lys-C Mix (Promega).

Peptides were analysed on the same LC–MS system and mobile phases as for intact mass analysis. Peptides were separated on a ACQUITY UPLC Peptide CSH C18, 130 Å, 1.7 µm, 2.1 × 150 mm applying a 25 min gradient from 5–30% B, increasing further to 95% B within 5 min, resulting in total run time of 44 min, with a flow rate of 0.25 mL/min. Electrospray ionization was performed in positive ionization mode, the resolution was set to 120 000 (at 200 m/z), with a scan range of 200–2000 m/z for MS1 analysis. A Top N method was applied for fragmentation with CID Assisted Collision and resulting fragments analysed in the Orbitrap at a resolution of 30 000 (at 200 m/z).

The raw data files were subjected to the Byos software (v 4.2) from Protein Metrics Inc. for data processing and reporting. For intact mass evaluation, peaks found in the total ion chromatogram were integrated and full mass spectra were deconvoluted. For DSB analysis, the Byos DSB workflow was used, searching against a built-in database based on the sequence of the POI.

### *1D-*^*1*^*H-NMR analysis*

Samples were dissolved in a buffer containing 20 mM Phosphate, 20 mM NaCl, at pH 5.5 and were analysed by 1D-^1^H-NMR after extensive dialysis in a common pool of buffer to reduce effects due to any systematic differences in sample preparation. Before NMR spectra were collected 5% D_2_O was added as a lock solvent. Spectra were acquired at 25 ℃ using a double pulse field gradient spin echo sequence (DPFGSE) to suppress water [15] on a Bruker Advance III spectrometer operating at 600 MHz with a He cooled QCI-P cryogenic probe using Topspin 3.6.1. Spectra were measured using 32 k complex data points over a sweep width of 9615 Hz using 1024 scans with an inter-scan relaxation delay of 1 s and 4 dummy scans for equilibration. These data were processed and analysed using Topspin and an exponential window function of 3 Hz was applied to improve signal to noise.

## Supplementary Information

**Additional file 1. Figure S1**. scFvM purification via two steps chromatography. **A** Representative Coomassie blue-stained gel of the scFvM produced with W3110 and purified via IMAC. The samples analysed are the total protein sample loaded (L), column flow-though (FT), eluates (E) from either the periplasm or the culture supernatant and the purified protein after dialysis (D) in non-reducing condition. Ladder (Mark12^TM^ Unstained Standard) on the left in kDa. **B** Representative Coomassie blue-stained gel of a CEX run in non-reducing condition on the scFvM produced with the W3110 strain. peak N°2 elution fractions are in the continued box while peak N°5 ones are in the dotted box.  

## Data Availability

The data used and /or analysed during the current study are available from the corresponding authors on reasonable requests.

## Abbreviations

CEX: Cation exchange chromatography
DO: Dissolved oxygen
DoE: Design of experiment
DSB: Disulphide bond
DSC: Differential scanning calorimetry
IB: Inclusion body
IMAC: Immobilised metal affinity chromatography
IPTG: Isopropyl β-D-1-thioglactopyranoside
MF: Multifermenter
MS: Mass spectrometry
MWCO: Molecular weight cut-off
NMR: Nuclear magnetic resonance
OD: Optical density
PCN: Plasmid copy number
POI: Protein of interest
PTM: Post-translational modification
scFv: Single-chain variable fragment
SP: Signal peptide
