# Supplementary material for: Yields and product comparison between Escherichia coli BL21 and W3110 in industrially relevant conditions: anti-c-Met scFv as a case study
Source: Microb Cell Fact. 2023 May 19;22:104. doi: 10.1186/s12934-023-02111-4 (PMC10197847; doi:10.1186/s12934-023-02111-4)
Supplement: Supplementary file 1 — Additional file 1. Figure S1. scFvM purification via two steps chromatography. A Representative Coomassie blue-stained gel of the scFvM produced with W3110 and purified via IMAC. The samples analysed are the total protein sample loaded (L), column flow-though (FT), eluates (E) from either the periplasm or the culture supernatant and the purified protein after dialysis (D) in non-reducing condition. Ladder (Mark12TM Unstained Standard) on the left in kDa. B Representative Coomassie blue-stained gel of a CEX run in non-reducing condition on the scFvM produced with the W3110 strain. peak N°2 elution fractions are in the continued box while peak N°5 ones are in the dotted box. [file 12934_2023_2111_MOESM1_ESM.docx]

Additional file


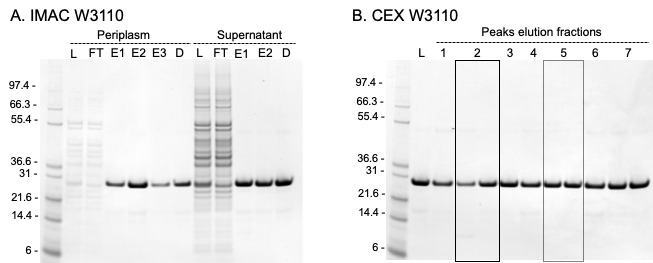


*Figure S1*. ***scFvM purification via two steps chromatography****. A) Representative Coomassie blue-stained gel of the scFvM produced with W3110 and purified via IMAC. The samples analysed are the total protein sample loaded (L), column flow-though (FT), eluates (E) from either the periplasm or the culture supernatant and the purified protein after dialysis (D) in non-reducing condition. Ladder (Mark12^TM^ Unstained Standard) on the left in kDa. B) Representative Coomassie blue-stained gel of a CEX run in non-reducing condition on the scFvM produced with the W3110 strain. peak N°2 elution fractions are in the continued box while peak N°5 ones are in the dotted box.*
